# Inhibition of porcine reproductive and respiratory syndrome virus replication by rifampicin *in vitro*

**DOI:** 10.3389/fvets.2024.1439015

**Published:** 2024-07-10

**Authors:** Ruiping Wei, Lu Li, Haifan Chen, Xiaoying Wang, Yaosheng Chen, Xiaohong Liu

**Affiliations:** State Key Laboratory of Biocontrol, School of Life Sciences, Sun Yat-sen University, Guangzhou, Guangdong, China

**Keywords:** rifampicin, antiviral drugs, PRRSV, viral replication, PEDV, PEAV

## Abstract

Porcine reproductive and respiratory syndrome virus (PRRSV) continues to cause significant economic losses to the global swine industry, yet effective prevention and control measures remain elusive. The development of novel antivirals is thus urgently needed. Rifampicin (RFP), a semisynthetic derivative of rifamycin, has been previously reported to inhibit the replication of certain mammalian DNA viruses as well as RNA viruses. In this study, we unveil RFP as a potent inhibitor of PRRSV both in Marc-145 cells (half-maximal inhibitory concentration 61.26 μM) and porcine alveolar macrophages (half-maximal inhibitory concentration 53.09 μM). The inhibitory effect of RFP occurred during viral replication rather than binding, internalization and release. We also demonstrated that RFP inhibits PRRSV proteins production in the early stage of infection, without inhibiting host protein synthesis. Moreover, RFP effectively restricted porcine epidemic diarrhea virus (PEDV) and porcine enteric alphacoronavirus (PEAV) infection in Vero cells. In summary, these findings indicate the promising potential of RFP as a therapeutic agent for PRRSV, PEDV and PEAV infection in pig farms.

## 1 Introduction

Porcine reproductive and respiratory syndrome (PRRS) has emerged as a widespread and highly contagious disease, characterized by reproductive failure in sows and respiratory ailments in piglets and growing pigs (1, 2). For over three decades, PRRS has posed a significant threat to global pork production since the first outbreak in the late 1980s (3). The etiological agent of PRRS is the PRRS virus (PRRSV), an enveloped, single-stranded, positive-sense RNA virus with a genome of approximately 15 kb, encoding at least 11 open reading frames (ORFs) (4). ORF1a and ORF1b, comprising about 75% of the full-length genome, encode two polyproteins (pp1a and pp1ab) which are processed into 14 functional nonstructural proteins (nsps) through a complex proteolytic cascade (4). Distinct subgenomic RNAs drive the expression of structural proteins, including glycosylated membrane proteins GP2, E, GP3, GP4, GP5, a non-glycosylated membrane protein (M), and the nucleocapsid (N) protein (5). The current control strategies, which primarily depend on vaccines, public health interventions, or genetically modified swine, indicates that achieving eradication of PRRSV remains a formidable challenge (6). Therefore, developing effective and safe strategies to control PRRSV infection is urgently needed.

Rifampicin, a semisynthetic ansa-macrolide, is known for its distinctive basket-like structure, which is formed by a structural motif called “ansa-bridge” (7). RFP is highly effective in treating bacterial infections, particularly in drug-sensitive tuberculosis, because of its ability to inhibit bacterial RNA polymerase (8–10). Recent studies have evaluated RFP and its derivatives as potential therapeutic agents against SARS-CoV-2, due to their ability to inhibit the activity of the viral RNA-dependent RNA polymerase (11, 12). In addition, RFP has been reported to exhibit inhibitory effects on a variety of viruses, including Moloney sarcoma virus, African swine fever virus, Rous sarcoma virus, influenza A virus, and vaccinia virus (13–18). However, it remains unknown whether RFP is effective in preventing and controlling PRRSV infection.

In this study, we explored the antiviral effects of RFP on PRRSV infection and further explored its antiviral mechanisms *in vitro*. We found that RFP effectively inhibited PRRSV replication in both Marc-145 cells and porcine alveolar macrophages (PAMs). RFP restrained PRRSV infection by preventing viral RNA and protein synthesis without suppressing host protein synthesis. Additionally, RFP can inhibit PEDV and PEAV infection in Vero cells. Taken together, RFP is highly valuable in clinical applications and could be helpful for therapies against viral infections in pig farms.

## 2 Materials and methods

### 2.1 Ethics statement

The sampling of primary porcine pulmonary alveolar macrophages derived from one-month-old pigs was conducted in accordance with the guidelines outlined in the Guide for the Care and Use of Laboratory Animals and Laboratory Animal Requirements for Environment and Housing Facilities (GB14925-2010/XG1-2011, National Laboratory Animal Standardization Technical Committee). The research protocol associated with this process was approved under license number IACUC-DD-16-0901 by the Institutional Animal Care and Use Committee (IACUC) of Sun Yat-sen University.

### 2.2 Chemicals, cells and virus

Rifampicin (M5930-100 mg) was purchased from Abmole. PAMs were isolated from one-month-old specific-pathogen-free piglets and were cultured at 37°C in 5% CO2 in RPMI-1640 medium supplemented with 10% fetal bovine serum (FBS) and 2% Penicillin–Streptomycin Solution (Fdbio Science, China). Marc-145 cells and Vero cells were cultured in Dulbecco’ s modified Eagle’ s medium (DMEM) (Corning, United States) with 10% FBS at 37°C in 5% CO2. Two PRRSV strains, GDBY1 (PRRSV-2), and JXA-1 (PRRSV-2), were provided by Dr. Heng Wang from South China Agricultural University and propagated in Marc-145 cells, titrating to a 50% tissue culture infective dose (TCID50). The PEDV and PEAV were provided by Dr. Yongchang Cao from Sun Yat-Sen University and propagated in Vero cells.

### 2.3 Detection of cytotoxicity of RFP

PAMs, Marc-145 cells, and Vero cells were seeded into 96-well plates at a density of 5,000 cells per well. Subsequently, different concentrations of RFP (0, 10, 25, 50, 75, 100, 125, 150, 175, 200 μM) were added to each well, followed by 24-h of incubation. The medium was then removed, and the cells were washed with PBS three times. Fresh DMEM containing 10% FBS and 10% CCK-8 solution (YEASEN, China) was added, followed by an additional 1.5-h incubation. Absorbance at 450 nm was measured using a microplate reader (BioTek, United States) to determine the half cytotoxic concentration (CC50) from the cell viability curve.

### 2.4 Antibodies

The following antibodies were used for western blot and immunoprecipitation analysis: anti-PRRSV N (4A5) antibody (9041) was purchased from MEDIAN Diagnostics (MEDIAN, Republic of Korea). Anti-PRRSV nsp4 protein antibody (GTX133700) and anti-GAPDH antibody (GTX627408) were purchased from GeneTex (United States). Anti dsRNA antibody J2 (10010200) was purchased from Scicon (Hungary). Anti-PRRSV nsp2 antibody was provided by Dr. Hanchun Yang from China Agricultural University. Anti-PEDV N protein antibody and ant-PEAV N protein antibody were provide by Dr. Yongchang Cao from Sun Yat-Sen University.

### 2.5 PRRSV infection and viral titration assays

Marc-145 cells were seeded 1 day before infection. Following a 2-h inoculation with PRRSV, the inoculum was removed, and the cells were washed once with PBS. Subsequently, fresh medium supplemented with 2% FBS was added. To determine the titer in the culture supernatant, the supernatant was serially diluted 10-fold and then inoculated in quadruplicate into Marc-145 cells seeded in 96-well plates. The cells were cultured for 4 days, and the wells showing cytopathic effects were recorded. The titer was calculated using the Reed–Muench method (19).

### 2.6 Viral attachment, internalization, replication and release assays

For viral attachment assay, Marc-145 cells were seeded in twelve-well plates. The control groups were treated with DMSO instead of RFP. Cells were infected with GDBY1 (MOI = 10) and incubated in a medium containing 150 μM RFP at 4°C for 2 h. Then, the cells were washed three times with pre-cooled PBS and then lysed to assess the expression of viral proteins.

For the internalization assay, Marc-145 cells were exposed to GDBY1 (MOI = 10) for 2 h at 4°C. Following the binding of virus particles to the cell surface, the cells underwent three washes with pre-cooled PBS and were then treated with a medium containing 150 μM RFP for 2 h at 37°C. Subsequently, the cells were washed three times with PBS, and 300 μL of protease K was added to each well for 30 min incubation at 4°C. After three additional washes with pre-cooled PBS, the cells were lysed for western blot analysis.

To investigate the effects of RFP on viral replication, Marc-145 cells were infected with GDBY1 at an MOI of 3 and treated with 150 μM RFP at indicated time points. At 12 hpi, the cells were lysed for western blot.

In the release assay, cells were incubated with GDBY1 (MOI = 3) at 37°C for 1 h. After virus adsorption, the cells underwent three washes with PBS and fresh medium were added. At 12 hpi, RFP was added and fresh medium were added after 2 h incubation. The cell supernatants were then collected for viral titer titration assay.

### 2.7 Western blot

Cellular proteins were extracted at specified time points using cell lysis buffer (Beyotime, China) supplemented with phenylmethylsulfonyl fluoride (PMSF) (Beyotime, China) and phosphatase inhibitors. The lysates were then separated by 8–15% sodium dodecyl sulfate-polyacrylamide gel electrophoresis (SDS-PAGE) and transferred onto a polyvinyl difluoride (PVDF) membrane (Roche, United states) using a semi-dry transfer method. To block non-specific binding, the membrane was incubated in 5% bovine serum albumin (BSA) (Fdbio Science, China) in TBST buffer (20 mM Tris–HCl pH 8.0, 150 mM NaCl, 0.05% Tween 20) for 1.5 h at room temperature. Subsequently, the membranes were probed with primary antibodies overnight at 4°C, followed by incubation with corresponding secondary antibodies for 1 h at 37°C. Protein bands were visualized using enhanced chemiluminescent reagent (ECL) (Fdbio Science, China) following the manufacturer’s instructions.

### 2.8 Quantitative real-time reverse-transcription PCR

Total RNAs were extracted from cells using TRIzol reagent (Magen, China) following the manufacturer’s instructions. Reverse transcription of 1.0 μg of RNAs was performed using the Reverse Transcription System (A3500, Promega, United States). The reverse-transcription products were amplified using 2 × RealStar Green Power Mixture (GenStar, China) and QuantStudio 7 Flex (Applied Biosystems). GAPDH served as a housekeeping gene for normalization. The relative expression of target genes was determined using the 2^−ΔΔCt^ method, with normalization to the mean Ct value of GAPDH. The primer sequences used for qRT-PCR are provided below:

GAPDH-Fw: TGACAACAGCCTCAAGATCG;

GAPDH-Rv: GTCTTCTGGGTGGCAGTGA;

PRRSV-N-Fw: AAAACCAGTCCAGAGGCAAG;

PRRSV-N-Rv: CGGATCAGACGCACAGTATG;

PEDV-N-F: GGGTATTGGAGAAAATCCTGATAG,

PEDV-N-R: AACTGGCGATCTGAGCATAG,

PEAV-N-F: CTGACTGTTGTTGAGGTTAC,

PEAV-N-R: TCTGCCAAAGCTTGTTTAAC.

### 2.9 Immunofluorescence

Marc-145 cells were fixed with 4% paraformaldehyde for 10 min at room temperature. Following fixation, cells were permeabilized with 0.1% Triton X-100 in PBS for 15 min in preparation for immunostaining. The cells were then blocked with 1% BSA for 30 min before being incubated with primary antibodies overnight at 4°C. After thorough washing to remove excess antibodies, the cells were exposed to Alexa Fluor-conjugated secondary antibodies for 1 h in the dark with gentle shaking. Subsequently, staining with 4,6-diamidino-2-phenylindole dihydrochloride (DAPI) in PBS was carried out for 5 min. Fixed cells were imaged using either an inverted fluorescence microscope (Nikon Eclipse Ti2-U, Japan).

### 2.10 Statistical analysis

All results are given as mean ± SE and analyzed using statistical tools implemented in GraphPad Prism 8.0 software. Statistical analyses were conducted using two-tailed unpaired Student’s *t*-tests for two groups and ANOVA for multiple comparisons. The significance levels are ^*^p < 0.05, ^**^p < 0.01 and ^***^p < 0.001.

## 3 Results

### 3.1 RFP dose-dependently decreased PRRSV nucleocapsid protein levels

**Figure 1:**
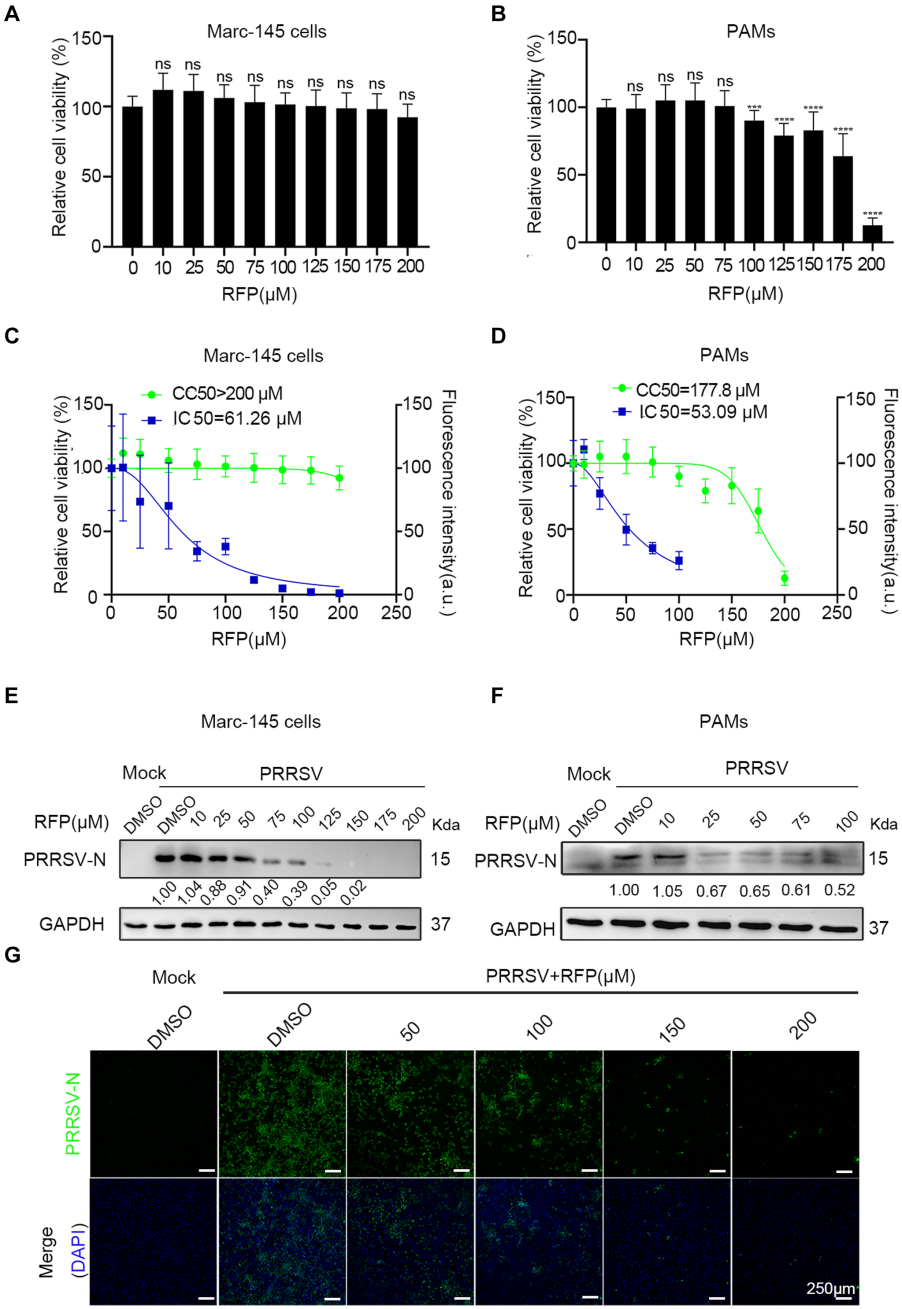
RFP dose-dependently decreased PRRSV nucleocapsid protein levels. **(A,B)** Marc-145 cells or PAMs were treated with the indicated concentrations of RFP for 24 h and the cell viability was examined by using the CCK8 kit. ^***^p < 0.001; ^****^p < 0.0001. **(C,D)** CC50 and IC50 were calculated using GraphPad Prism. **(E–G)** Marc-145 cells or PAMs were infected with PRRSV at an MOI of 0.1 after RFP treatment (indicated concentration). At 24 hpi, PRRSV replication was monitored by western blot **(E,F)** and immunofluorescence **(G)**.

We first determined the cytotoxic effect of RFP by obtaining a half maximal cytotoxic concentration (CC50). In Marc-145 cells, the CC50 exceeded 200 μM (Figures 1A,C), while in PAMs, it was approximately 177.8 μM (Figures 1B,D). Next, to assess the anti-PRRSV activity of RFP, we examined its ability to inhibit the expression of the PRRSV nucleocapsid (N) protein, which is the most abundant viral protein in infected cells. Western blot analysis revealed a dose-dependent reduction in viral N protein expression upon treatment with RFP in both Marc-145 cells and PAMs (Figures 1E,F). Immunofluorescence also showed that the number of cells with N-specific staining was significantly reduced in a dose-dependent manner by RFP (Figure 1G). In addition, we determined the half-maximal inhibitory concentration (IC50) of RFP, which was calculated to be 61.26 μM in Marc-145 cells and 53.09 μM in PAMs (Figures 1C,D). These findings indicate that RFP effectively reduced PRRSV N protein expression in a dose-dependent manner in both Marc-145 cells and PAMs.

### 3.2 RFP inhibited PRRSV proliferation *in vitro*

**Figure 2:**
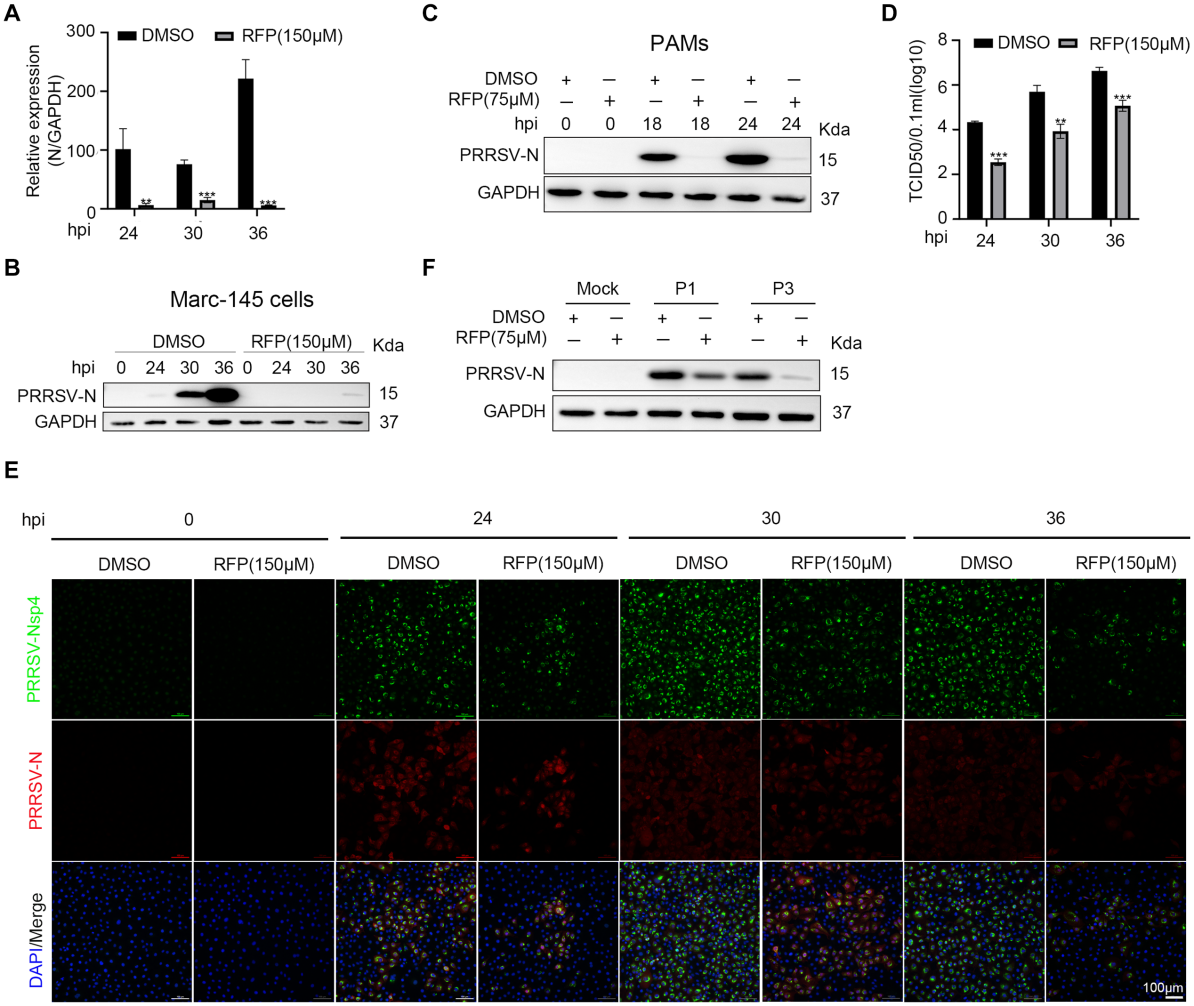
RFP inhibited PRRSV proliferation *in vitro*. **(A)** Marc-145 cells were infected with PRRSV at an MOI of 0.1 for the indicated periods after RFP (150 μM) treatment. The mRNA levels of PRRSV-N were measured by qRT-PCR. GAPDH serves as a control. ^**^p < 0.01; ^***^p < 0.001. **(B,C)** Marc-145 cells or PAMs were infected with PRRSV and treated with RFP, at 24 hpi, the PRRSV N protein levels were measured by western blot. **(D)** Marc-145 cells were infected with PRRSV at an MOI of 0.1 and treated with RFP, at the indicated time, viral production in cells was measured and is shown as TCID50. ^**^p < 0.01; ^***^p < 0.001. **(E)** The same as **(D)** except that immunofluorescence was used. **(F)** Marc-145 cells were, respectively, infected with three successive generations of screened viruses after RFP treatment, and the protein levels of PRRSV N were measured by western blot.

As the inhibition of PRRSV N protein expression by RFP, we further investigated the impact of RFP on viral proliferation and spread. As shown in Figures 2A–D, RFP treatment significantly reduced PRRSV N mRNA and protein levels, as well as viral titers, throughout the course of infection. Consistent with this, Immunofluorescence analysis showed that the number of infected cells were significantly reduced at indicated time points (Figure 2E). These findings suggest that RFP effectively inhibits PRRSV proliferation and dissemination *in vitro*.

The substantial genetic diversity within RNA virus populations increases the likelihood of acquiring drug resistance under strong selective pressure (20). Therefore, we investigated the possibility of resistance emergence in PRRSV infection. Marc-145 cells were continuously infected with PRRSV in a medium containing 150 μM RFP, resulting in three successive generations of screened viruses (P1, P2, and P3). We found that the replication of P1 or P3 was still inhibited by RFP, indicating that P1 or P3 did not develop resistance to RFP (Figure 2F).

### 3.3 RFP inhibited the replication stage of PRRSV

**Figure 3:**
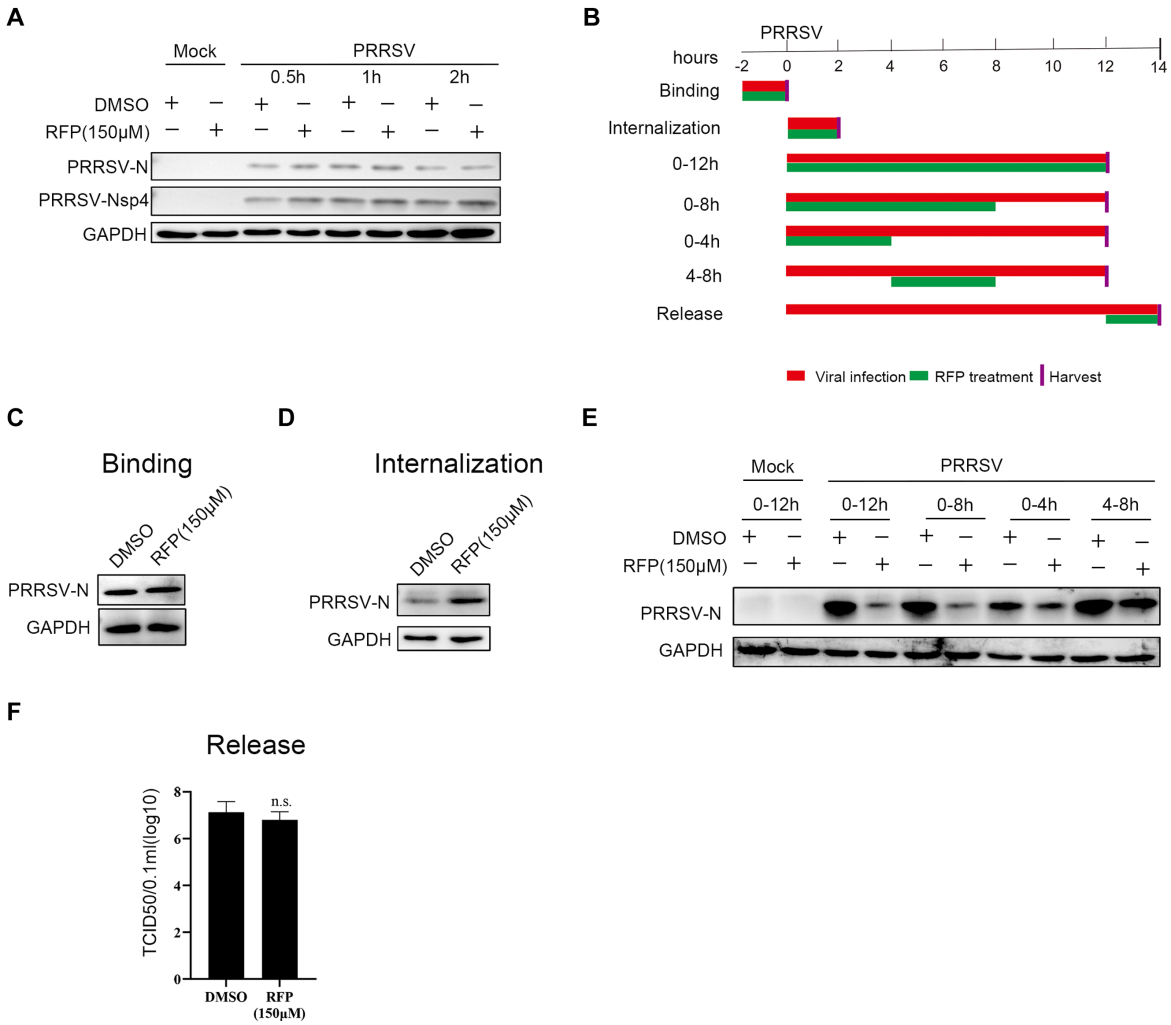
Effect of RFP on PRRSV life cycle. **(A)** PRRSV was incubated with RFP at the indicated concentration for 0.5, 1 or 2 h at 37°C, and then the virus were used to infect Marc-145 cells. At 24 hpi, The N and Nsp4 proteins of PRRSV were measured by western blot. **(B)** Schematic of the experimental approach of adding the drug at different time points. Marc-145 cells were infected with GDBY1, followed by addition of RFP at designated times. Red bars represent the PRRSV infection period, green bars represent RFP treatment. **(C–F)** Viral binding, internalization, replication, and release were performed as described in Material and Methods.

We have proved that RFP can impede the proliferation of PRRSV, but the precise mechanism underlying the antiviral activity of RFP against PRRSV remains elusive. Therefore, to assess whether RFP directly inactivates PRRSV particles, PRRSV was pre-incubated with RFP for 0.5 h, 1 h or 2 h in the incubator before transferring to monolayer Marc-145 cells. As a control, an equivalent volume of DMSO was added. At 24 hpi, the cells were collected for western blot analysis. The result showed that the expression of PRRSV early nonstructural protein 4 (nsp4) and N protein have no significantly change, indicating that RFP does not directly inactivate PRRSV particles (Figure 3A).

To investigate which stage of PRRSV infection is affected by RFP, we performed a time-of-addition assay in Marc-145 cells (Figure 3B). The early steps of PRRSV infection include binding to the cell surface and internalization into the cell. To investigate whether RFP affects PRRSV attachment. The N protein levels of attached viruses were quantified by western blot. We found that RFP treatment did not reduce virus binding (Figure 3C), indicating that RFP has no effect on PRRSV binding.

As for PRRSV internalization affected by RFP, the N protein levels of internalized viruses were quantified by western blot. The N protein levels inside the RFP-treated cells did not reduce compared to DMSO-treated cells (Figure 3D), indicating that RFP has no inhibitory effect on virus internalization. However, RFP exhibited potent inhibition of the replication phase, leading to a significant reduction in viral N protein levels (Figure 3E). Regarding viral release, the quantification of PRRSV release was conducted by measuring the number of infectious virus particles in the supernatant. As showed in Figure 3F RFP did not hinder PRRSV release, which was assessed through viral titer analysis. Based on these findings, we conclude that RFP inhibits PRRSV proliferation by specifically targeting the viral replication stage.

### 3.4 RFP inhibits viral RNA and proteins production in the early stage of infection

**Figure 4:**
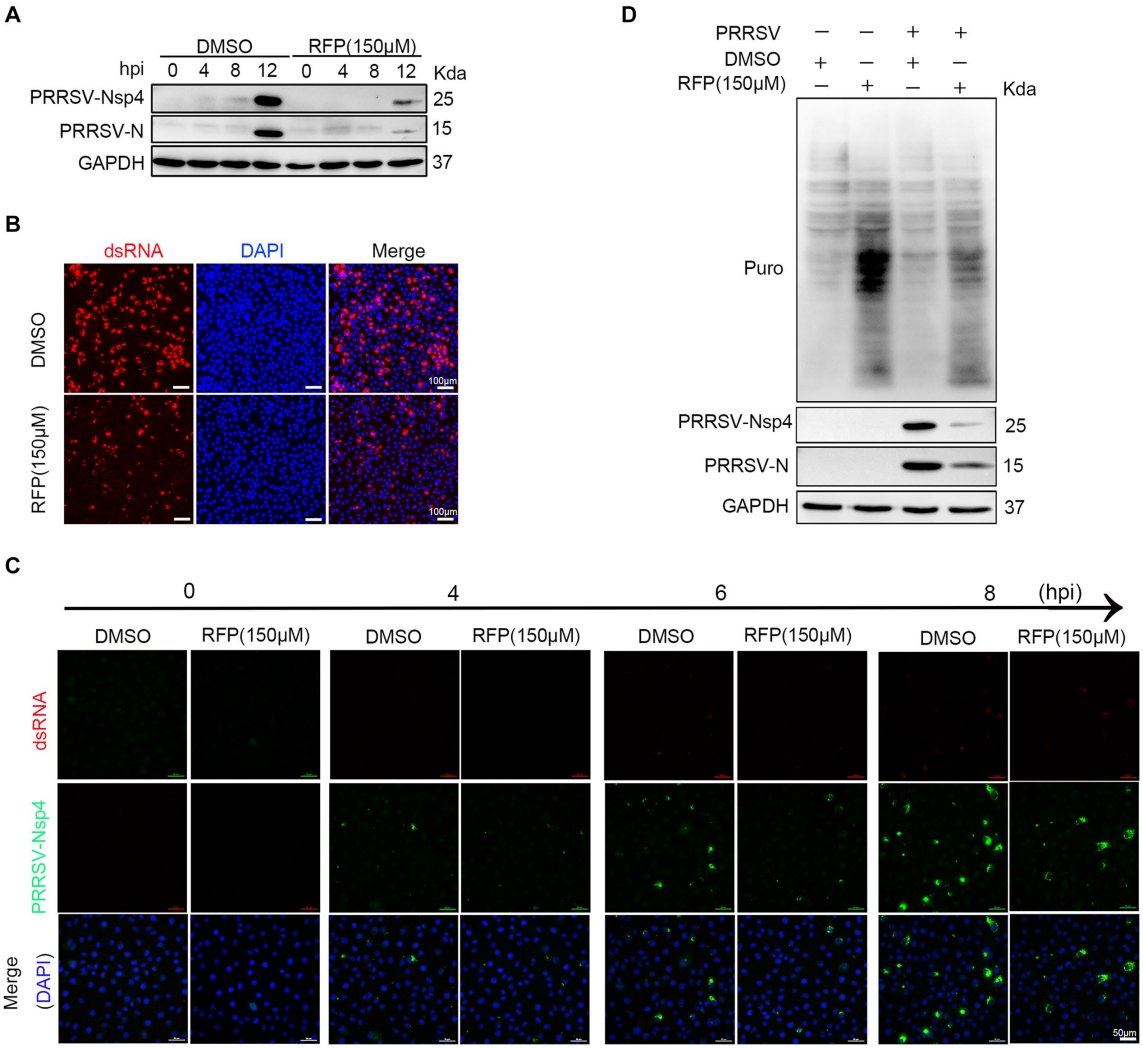
RFP inhibits viral RNA and proteins production in the early stage of infection. **(A)** Marc-145 cells were treated with RFP and infected with PRRSV at an MOI of 1 for the indicated periods. The levels of viral N and Nsp4 proteins were measured by western blot analysis. **(B)** Marc-145 cells were infected with PRRSV and treated with RFP, and at 24 hpi, the levels of dsRNA were assessed by immunofluorescence staining. **(C)** The same as **(A)**, except that immunofluorescence was conducted. **(D)** Marc-145 cells were treated with either RFP or DMSO and infected with PRRSV at an MOI of 1. At 23.5 hpi, puromycin dihydrochloride (Puro, 10 μg/mL) was added to the medium, and protein samples were collected after an additional 0.5 h of incubation. The level of newly synthesized total proteins in the cells was determined by western blot analysis using the Puromycin antibody (MABE343).

Previous study has shown that RFP can inhibit the activity of RNA-dependent RNA polymerase (RdRp) of SARS-CoV-2 (11). We hypothesized that RFP might have a similar effect on the RdRp of PRRSV, thereby affecting the synthesis of viral RNA and proteins. To test this, we examined the production of non-structural proteins (nsp2 and nsp4), double-stranded RNA (dsRNA), and structural protein (N protein) in the presence of RFP or DMSO during a single round of infection. As expected, treatment with RFP resulted in a reduction in dsRNA and N protein production at the early stage of infection, as well as nsp2 and nsp4 (Figures 4A–C). These findings suggest that RFP inhibits the synthesis of viral RNA and proteins in the early stages of infection.

Viruses, as obligate intracellular parasites, rely exclusively on the host translation machinery for protein synthesis (21). However, it is still uncertain whether RFP inhibits PRRSV protein production by reducing overall protein synthesis in host cells. To investigate this, we quantified total protein synthesis in Marc-145 cells by western blot. The results show that RFP treatment did not impede host protein synthesis (Figure 4D). These results suggest that RFP may specifically suppress PRRSV protein synthesis at an early stage without affecting host protein synthesis.

### 3.5 RPF inhibited the replication of PEDV and PEAV

**Figure 5:**
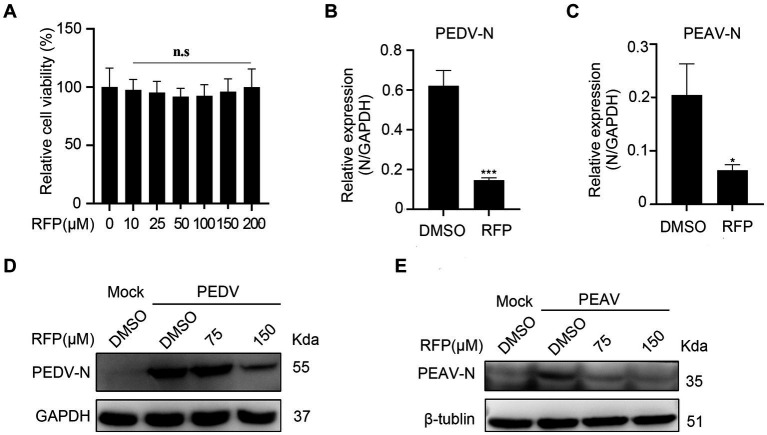
Inhibition the replication of PEDV and PEAV by RFP. **(A)** The cytotoxicity of the RFP on Vero cells were determined by CCK8 assays. **(B,C)** qRT-PCR was performed on Vero cells that were inoculated with PEDV at an MOI of 0.5 or PEAV at an MOI of 1.0, and treated with either DMSO or 150 μM for 8 or 12 h. **(D,E)** Similarly, western blot analysis was performed on Vero cells treated as described in **B**,**C**. ^*^p < 0.01; ^***^p < 0.001.

To evaluate whether the antiviral effect of RFP is specific to PRRSV. we investigated the effects of RFP on PEDV and PEAV, two emerging enteric viruses of swine that belong to order *Nidovirales* together with PRRSV. Vero cells were treated with RFP for 24 h, followed by infection with PEDV or PEAV at an MOI of 0.5 and 0.1, respectively. The mRNA level of PEDV N or PEAV N were significantly reduced in RFP-treated cells compared to the control group (Figures 5B,C). Consistent with this result, the levels of N proteins for both viruses were also decreased in cells treated with RFP (Figures 5D,E). Importantly, cell viability remained unaffected at the dosage of RFP used in the experiment (Figure 5A). These findings demonstrate that RFP can also inhibits PEDV and PEAV infections in Vero cells, suggesting a potential broad-spectrum antiviral ability of RFP.

## 4 Discussion

PRRSV is recognized as one of the most economically damaging diseases of pigs globally (3). Initially reported in North America in the late 1980s, PRRSV rapidly disseminated to numerous countries, posing a huge challenge to the prevention of swine diseases in China (22). PRRSV induces a delayed onset and low titers of neutralizing antibodies, triggering a multifaceted evasion of the innate immune response (23, 24). Moreover, the continuous evolution of PRRSV strains and the occurrence of recombination between vaccine and wild-type strains result in suboptimal vaccine efficacy, making the control of PRRSV infection more challenging (25, 26). Previous studies have shown that treatments such as chemical compounds, herbal extracts, siRNA, microRNA and neutralizing antibodies can inhibit the replication of PRRSV *in vitro* (25, 27–29). However, these agents are still far from being used in animal husbandry. Despite persistent efforts, the prevention and treatment of PRRSV remain elusive. In this study, we found that RFP has a strong inhibitory effect against PRRSV infection without inducing cytotoxic effects in Marc-145 cells and PAMs. This indicates that RFP may serve as a promising new antiviral treatment for PRRSV.

In antiviral assay, PRRS viral replication, rather than binding, internalization or release, was strongly inhibited when Marc-145 cells or PAMs were treated with RFP (Figure 1), indicating that RFP might affect virus by impairing RNA and protein synthesis. Importantly, we have shown that RFP suppresses the production of viral RNA and proteins during the early stages of infection, without affecting host protein synthesis (Figure 4). This suggests that RFP inhibitory effects may be virus specific. It has been shown previously that RFP and its derivatives can inhibit the RdRp activity of SARS-CoV-2 *in vitro* (11, 12). We speculate that the inhibition of PRRSV infection by RFP might be attributed to the suppression of viral RdRp, leading to reduced production of virus RNA and proteins. However, this interpretation is speculative and requires further investigation.

Similar as PRRSV, despite intensive control measures, PEDV continues to impose significant economic burdens on swine farms (30). PEAV, a newly identified porcine enteric alphacoronavirus, is the first coronavirus to spread from bats to pigs (31). Unfortunately, there are no vaccines or specific antiviral drugs available to treat these two types of alphacoronaviruses infection currently. In this study, it was observed that RFP demonstrates inhibitory effects on PEDV and PEAV replication in Vero cells (Figure 5). Therefore, RFP shows promise as a potential therapeutic agent for future applications in combating PEDV and PEAV infections. This also indicates that RFP may be a broad-spectrum antiviral drug.

In conclusion, our study illustrates that RFP inhibits the infection and replication of PRRSV by targeting viral RNA and protein production in the early stages of infection. Additionally, RFP demonstrated suppression of PEDV and PEAV infection in Vero cells. These findings suggests RFP has the potential to be antiviral pharmaceuticals in future defenses against PRRSV, PEDV, and PEAV epidemics.

## Data availability statement

The original contributions presented in the study are included in the article/supplementary material, further inquiries can be directed to the corresponding author.

## Ethics statement

The animal study was approved by the Institutional Animal Care and Use Committee (IACUC) of Sun Yat-sen University. The study was conducted in accordance with the local legislation and institutional requirements.

## Author contributions

RW: Formal analysis, Investigation, Methodology, Resources, Software, Validation, Writing – review & editing. LL: Investigation, Methodology, Resources, Software, Validation, Writing – review & editing. HC: Investigation, Resources, Writing – original draft. XW: Investigation, Resources, Writing – review & editing. YC: Funding acquisition, Writing – review & editing. XL: Conceptualization, Data curation, Funding acquisition, Project administration, Writing – original draft.
